# Association and mediation between circulating inflammatory proteins and skin fibrosis

**DOI:** 10.3389/fendo.2025.1416993

**Published:** 2025-03-18

**Authors:** Zirui Zhao, Dongming Lv, Ruixi Zeng, Yanchao Rong, Zhongye Xu, Rong Yin, Zhicheng Hu, Xiaoling Cao, Bing Tang

**Affiliations:** ^1^ Department of Plastic Surgery, First Affiliated Hospital of Sun Yat-sen University, Guangzhou, Guangdong, China; ^2^ Department of Burns, Wound Repair and Reconstruction, First Affiliated Hospital of Sun Yat-sen University, Guangzhou, Guangdong, China; ^3^ Department of Dermatology, First Affiliated Hospital of Sun Yat-sen University, Guangzhou, Guangdong, China

**Keywords:** circulating inflammatory protein, skin fibrosis, bidirectional Mendelian randomization, mediation analysis, blood metabolite

## Abstract

**Objective:**

Skin fibrosis is a dermal lesion associated with inflammatory factors. However, the exact causal relationship between circulating inflammatory proteins (CIPs) and skin fibrosis remains unclear. To investigate this potential association and mediated effect, Mendelian randomization (MR) and two-step MR were used.

**Methods:**

Summary statistics from genome-wide association studies (GWAS) were extracted from the GWAS Catalog for CIPs, blood metabolites (BMs), and skin fibrosis. Two-sample MR and reverse MR were conducted to determine the effect of CIPs on skin fibrosis. Two-step MR was then performed to investigate the role of BMs in mediating the effect of CIPs on skin fibrosis. Reverse MR analysis was performed to confirm the unidirectional causality between CIPs and BMs, as well as between BMs and skin fibrosis.

**Results:**

Bidirectional Mendelian randomization revealed negative associations between skin fibrosis and the levels of T-cell surface glycoprotein CD6 isoform (odds ratio [OR] 0.670 [95% confidence interval [CI] 0.472, 0.951], *p* = 0.025), Delta and Notch-like epidermal growth factor-related receptor (OR 0.779 [95% CI 0.609, 0.998], *p* = 0.048), and Interleukin-10 receptor subunit beta (OR 0.541 [95% CI 0.332, 0.884], *p* = 0.014). There was a positive association between skin fibrosis and levels of Fibroblast growth factor 21 (OR 2.276 [95% CI 1.064, 4.870], *p* = 0.034). Two-step MR showed that Retinol (Vitamin A) to the linoleoyl-arachidonoyl-glycerol ratio (β_M_ 0.108 [95% CI 0.006, 0.210], *p* = 0.004) and the Cholesterol to linoleoyl-arachidonoyl-glycerol ratio (β_M_ 0.238 [95% CI 0.002, 0.474], *p* = 0.048) were identified as mediators, which showed evidence of the mediated effect of the levels of Fibroblast growth factor 21 on Keloid through these mediators.

**Conclusion:**

The study presented credible evidence of a causal association between CIPs and skin fibrosis, with BMs potentially acting as a mediator in this association. These findings offer new insights into early screening and prevention of skin fibrosis.

## 1 Introduction

Skin fibrosis refers to a group of skin conditions where there is an excessive deposition of connective tissue components in the dermis. It can be caused by damage from burns, surgery, or trauma, which disrupts the balance between extracellular matrix synthesis and degradation, leading to disease. Skin fibrosis, including keloid and hypertrophic scars, can cause a loss of physiological architecture and skin malfunction, resulting in physical and psychological distress for patients (1, 2). Keloid is a refractory skin fibrotic disease with a recurrence rate of up to 45%. It is commonly considered a benign skin tumor due to its aggressive proliferation, which can result in limited movement or disfigurement (3, 4). Early assessment and accurate treatment of diseases are crucial. However, our current understanding of diseases is imperfect, and efficient and rapid diagnostic methods are lacking. Therefore, it is necessary to deepen our knowledge and explore new diagnosis, prevention, and intervention methods.

Circulating inflammatory proteins (CIPs) plays an important role in many diseases. For example, IL-10 has been demonstrated to be implicated in cerebral microcirculatory defects and cognitive impairment associated with type 1 diabetes (5). High plasma levels of MIP-1β and TNF-α were positively related to atherosclerotic plaques with high inflammatory activity (6). Investigating the correlation between CIPs and diseases is essential for clarifying disease mechanisms and developing disease prevention and treatment strategies (7). TGFB is known to play crucial roles in the occurrence and development of skin fibrosis, as evidenced by their increased content in fibrotic skin tissue (8). However, there are no significant differences in serum levels of TGFB between patients with skin fibrosis and healthy individuals (9). The causal relationship between CIPs and skin fibrosis is still unclear.

Blood metabolite (BMs) is a small molecule of metabolic reaction. Serum BMs levels are influenced by various factors, such as genetics and diseases. Furthermore, they can affect diseases and serve as a therapeutic target (10, 11). For example, branched-chain amino acids were identified as insulin analogues. The high levels of branched-chain amino acids could eventually lead to insulin resistance and diabetes (12). Currently, researchers have found that metabolites might be associated with skin fibrosis. For example, butyrate is shown to improve skin fibrosis in mouse models (13). Furthermore, a decrease in L-tryptophan was identified in patients suffering from systemic sclerosis. The level of L-tryptophan in these patients exhibited a negative correlation with inflammatory markers, such as IL-6 (14). However, the causal relationship remains unclear. Determining the causal role of BMs in skin fibrosis can identify effective intervention points for therapies.

Genome-wide association studies (GWAS) contribute significantly to our understanding of skin fibrosis in genetics (7). With single nucleotide polymorphisms (SNPs) of GWAS, we can perform Mendelian randomization (MR) analysis to infer a credible causal relationship (15). As a widely used analytical method, MR can help reduce bias and eliminate reverse causality. This is because genetic variations are randomly assigned during meiosis and are independent of environmental and other acquired factors (16). Compared to traditional observational approaches, MR analysis does not require unmeasured confounding between exposure and outcome. Mediation analysis shares these strengths (17). MR studies revealed a causal effect of CIPs on diseases, such as osteoarthritis and colorectal cancer (18, 19). Currently, there is no MR evidence to establish a causal association or mediated effect between CIPs and skin fibrosis.

GWAS summary data from the GWAS Catalog were utilized to perform MR and reverse MR analyses to examine the causal relationship between CIPs and skin fibrosis. In addition, a two-step MR (also referred to as network MR) analysis was conducted to investigate the causal role of BMs in linking the effect of CIPs on skin fibrosis. These findings may guide the exploration of mechanisms and generate new ideas for reducing the risk of skin fibrosis.

## 2 Materials and methods

### 2.1 Design of study

**Figure 1:**
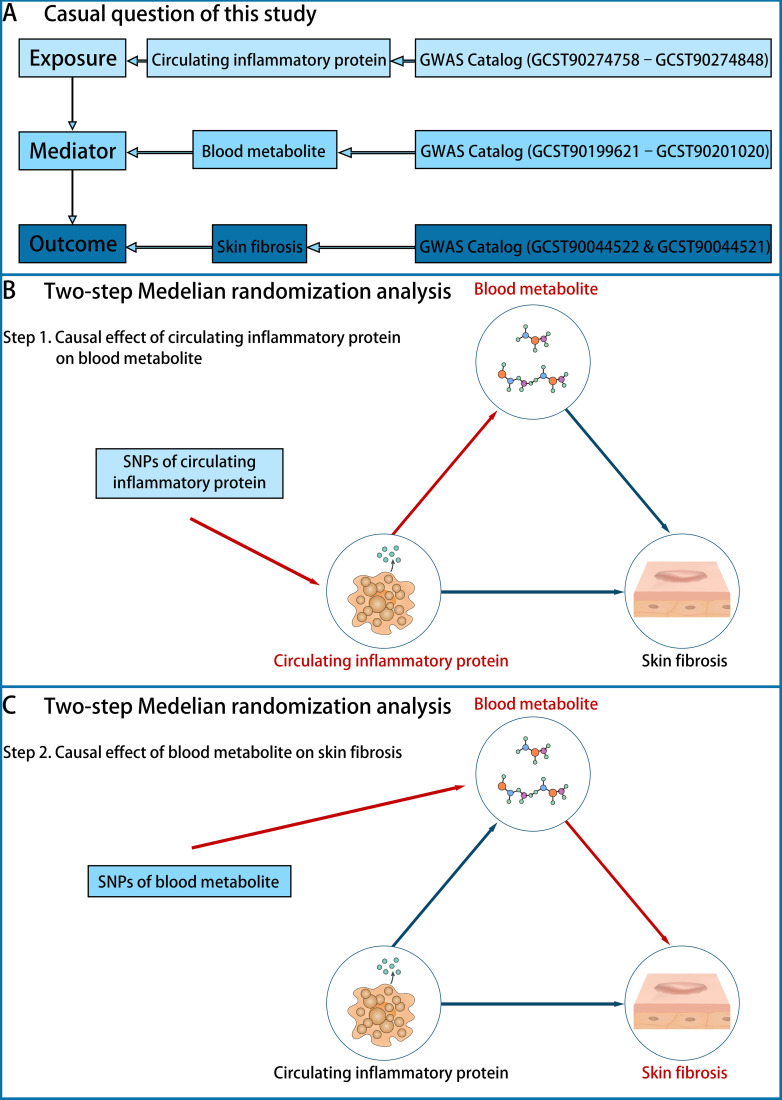
The design of the study. **(A)** The question is whether there is a causal role of BMs in mediating the effect of CIPs (exposure) on skin fibrosis (outcome). The data on CIPs was extracted from the GWAS Catalog (GCST90274758–GCST90274848), the data on BMs was extracted from the GWAS Catalog (GCST90199621–GCST90201020) and the data on skin fibrosis was extracted from the GWAS Catalog (GCST90044522 & GCST90044521). **(B)** Step 1 of the two-step of MR model: establishing the causal effect of CIPs (exposure) on BMs (mediator). **(C)** Step 2 of the two-step of MR model: establishing the causal effect of BMs (mediator) on skin fibrosis (outcome).

Two-sample MR and inverse MR analyses were performed on datasets from the GWAS catalog to determine the causal relationship between CIPs (exposure), BMs (mediator), and skin fibrosis (outcome) (**Figure 1A**). In step 1 of two-step of MR model, we established the causal effect of CIPs (exposure) on BMs (mediator) (**Figure 1B**). Then in step 2 of two-step MR model, we established the causal effect of BMs (exposure) on skin fibrosis (outcome) (**Figure 1C**). In the end, the mediated effect was calculated to determine the role of BMs in mediating the effect of CIPs on skin fibrosis (20, 21).

### 2.2 Data preparation

The GWAS Catalog (GCST90274758–GCST90274848) was used to extract summary statistics of CIPs traits, which encompass 91 inflammation-related plasma proteins. All CIPs were listed in the “CIPs list” (**Supplementary Table 1**). The data comprises 11 cohorts with a total of 14,824 participants of European ancestry (7). Summary statistics of BMs traits, including 1,091 blood metabolites and 309 metabolite ratios, were extracted from the GWAS catalog (GCST90199621-GCST90201020). The sample size consists of 8,299 individuals of European ancestry (10). All BMs were listed in the “BMs list” (**Supplementary Table 1**). The GWAS summary statistics for skin fibrosis traits from the GWAS Catalog (GCST90044522 & GCST90044521) include *keloid scar* and *Scar conditions and fibrosis of skin* (**Figure 1A**). GCST90044522 comprises 201 cases of European ancestry and 456,147 controls of European ancestry, while GCST90044521 comprises 1,887 cases of European ancestry and 454,461 controls of European ancestry (22). All skin fibrosis traits were listed in the “Skin fibrosis list” (**Supplementary Table 1**). A list of CIPs, BMs, and skin fibrosis markers used for data extraction from GWAS in the study is also presented in **Supplementary Table 1**.

### 2.3 SNPs selection

For each trait, only SNPs that showed a strong association (*p* < 5 × 10^−7^) were considered as instrumental variables (IVs). To avoid weak IV bias, SNPs with an *F*-statistic < 10 were not defined as IVs. Additionally, clumping was performed with the EUR population reference (r^2^ < 0.01 and clump distance > 10000 kb) to eliminate linkage disequilibrium (LD). SNPs related to confounders were also excluded based on PhenoScanner V2. Finally, palindromic SNPs were either harmonized or excluded using ‘TwoSampleMR’ R package. After selection, the remaining SNPs were considered as IVs for MR and reverse MR analyses (23–25).

### 2.4 MR and reverse MR analyses

MR and reverse MR analyses between exposure and outcome, between exposure and mediator, and between mediator and outcome were performed by R (version 4.3.2) and R package TwoSampleMR package (version 0.5.10).

Inverse variance-weighted (IVW), MR-Egger, weighted median, simple mode, and weighted mode methods were used to determine the causal association. P-value > 5 × 10^−2^ showed statistically significant. IVW was considered the primary method because of providing a more robust estimation (17). MR-Pleiotropy Residual Sum and Outlier (MR-PRESSO) was utilized to remove outliers (26). In sensitivity analysis, Cochran’s test was performed to assess heterogeneity while Q statistic P-value > 5 × 10^−2^ showed no heterogeneity. MR-Egger test was performed to assess horizontal pleiotropy while the P-value > 5 × 10^−2^ showed no pleiotropy (27, 28). We assessed the horizontal pleiotropy by a leave-one-out analysis (23).

First, we performed MR and reverse MR analyses between CIPs and skin fibrosis to determine the causal effect of CIPs on skin fibrosis, referred to as the total effect (β) (17, 21). The CIPs traits with a causal association with skin fibrosis were used in step 1 of two-step of MR model.

Due to the significant BMs traits, we conducted a two-sample MR analysis to identify the BMs traits that may be related to skin fibrosis. These BMs traits were utilized in step 1 of two-step of MR model.

Then in step 1 of two-step of MR model, we established the causal effect of CIPs (exposure) on BMs (mediator) by MR and reverse MR analyses (20, 21). The study referred to the effect as the β1 effect. In step 2 of the two-step MR model, we used the BMs traits that have a causal association with CIPs. We performed MR and reverse MR analyses to determine the causal effect of BMs (mediator) on skin fibrosis (outcome), which is referred to as the β_2_ effect.

In the end, the mediated effect (β_M_) was calculated by the product of the coefficients method. The mediated effect (β_M_) = β – (β_1_ × β_2_).

## 3 Results

### 3.1 IVs selection

**Figure 2:**
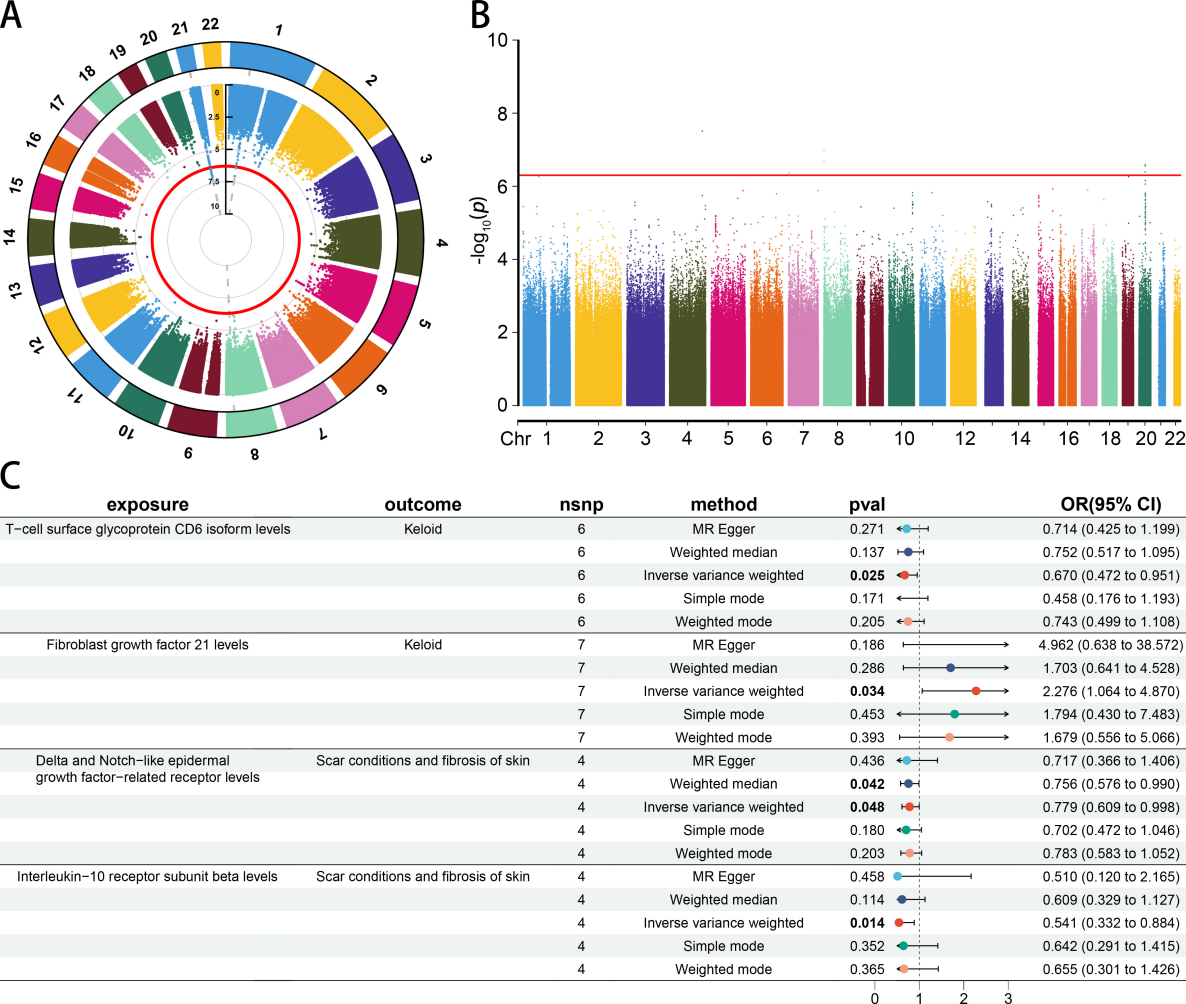
The causal association between CIPs and skin fibrosis. **(A)** The circle manhattan plot of the *Keloid scar* trait (GCST90044522) (threshold = 5 × 10^−7^). **(B)** The manhattan plot of the *Scar conditions and fibrosis of skin* trait (GCST90044521) (threshold = 5 × 10^−7^). **(C)** Forest plot of casual effect between CIPs and skin fibrosis.

Due to a lack of SNPs (*p* < 5 × 10^−8^) associated with skin fibrosis, we opted to extract SNPs with a P-value < 5 × 10^−7^ for further analysis (**Supplementary Table 2**). A circular manhattan plot was generated to display the chromosome positions and P-values of SNPs associated with the *Keloid scar* trait. The manhattan plot displayed the chromosome positions and P-value of SNPs associated with *Scar conditions and fibrosis of skin* trait. The red line represents the threshold line (*p* = 5 × 10^−7^) (**Figures 2A, B**).

### 3.2 Causal effects of the CIPs on skin fibrosis

The results of MR and reverse MR analyses indicated that T-cell surface glycoprotein CD6 isoform (CD6) levels were negatively associated with *Keloid* (odds ratio [OR] 0.670 [95% confidence interval [CI] 0.472, 0.951], *p* = 0.025). Fibroblast growth factor 21 (FGF21) levels were positively associated with *Keloid* (OR 2.276 [95% CI 1.064, 4.870], *p* = 0.034). Delta and Notch-like epidermal growth factor-related receptor (DNER) levels were negatively associated with *Scar conditions and fibrosis of skin* (OR 0.779 [95% CI 0.609, 0.998], *p* = 0.048). Interleukin-10 receptor subunit beta (IL1RB) levels were negatively associated with *Scar conditions and fibrosis of skin* (OR 0.541 [95% CI 0.332, 0.884], *p* = 0.014) (**Figure 2C**). More details could be found in **Supplementary Table 3**. A leave-one-out analysis, forest plot, and scatter plot were performed to verify the credibility of the results (**Supplementary Figures S1**-**4**).

### 3.3 Causal effects of the CIPs on BMs

**Figure 3:**
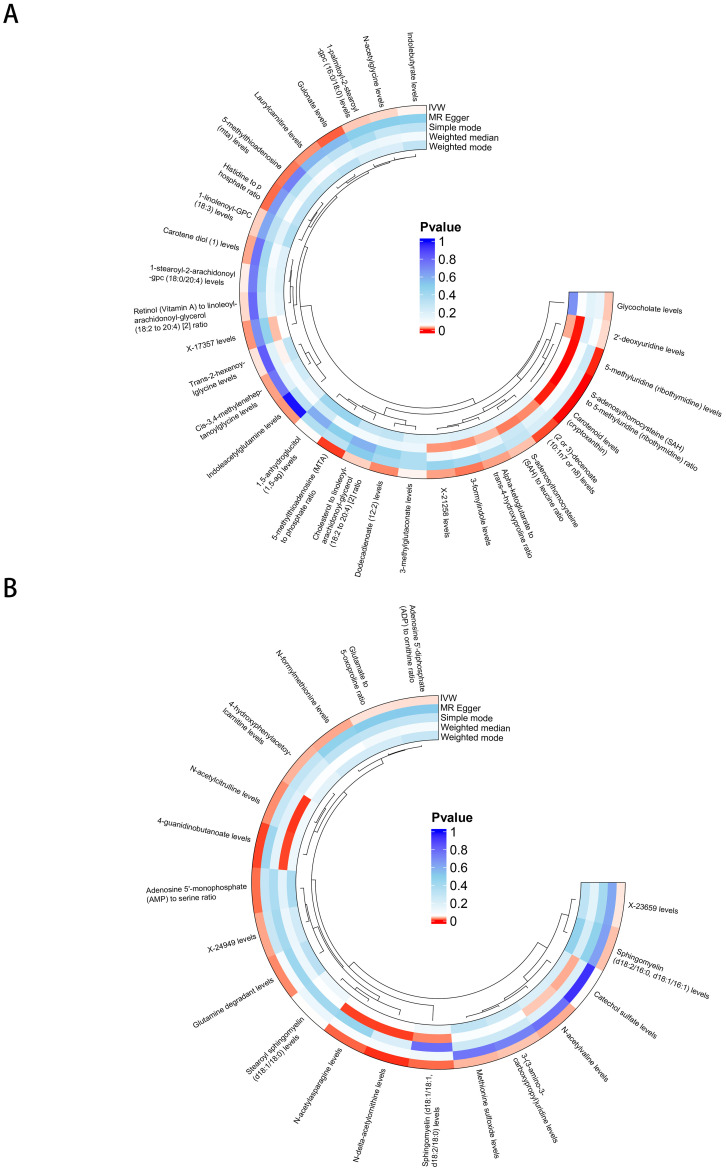
BMs potentially associated with skin fibrosis. **(A)** BMs related to *keloid*. **(B)** BMs related to *Scar conditions and fibrosis of skin*.

**Figure 4:**
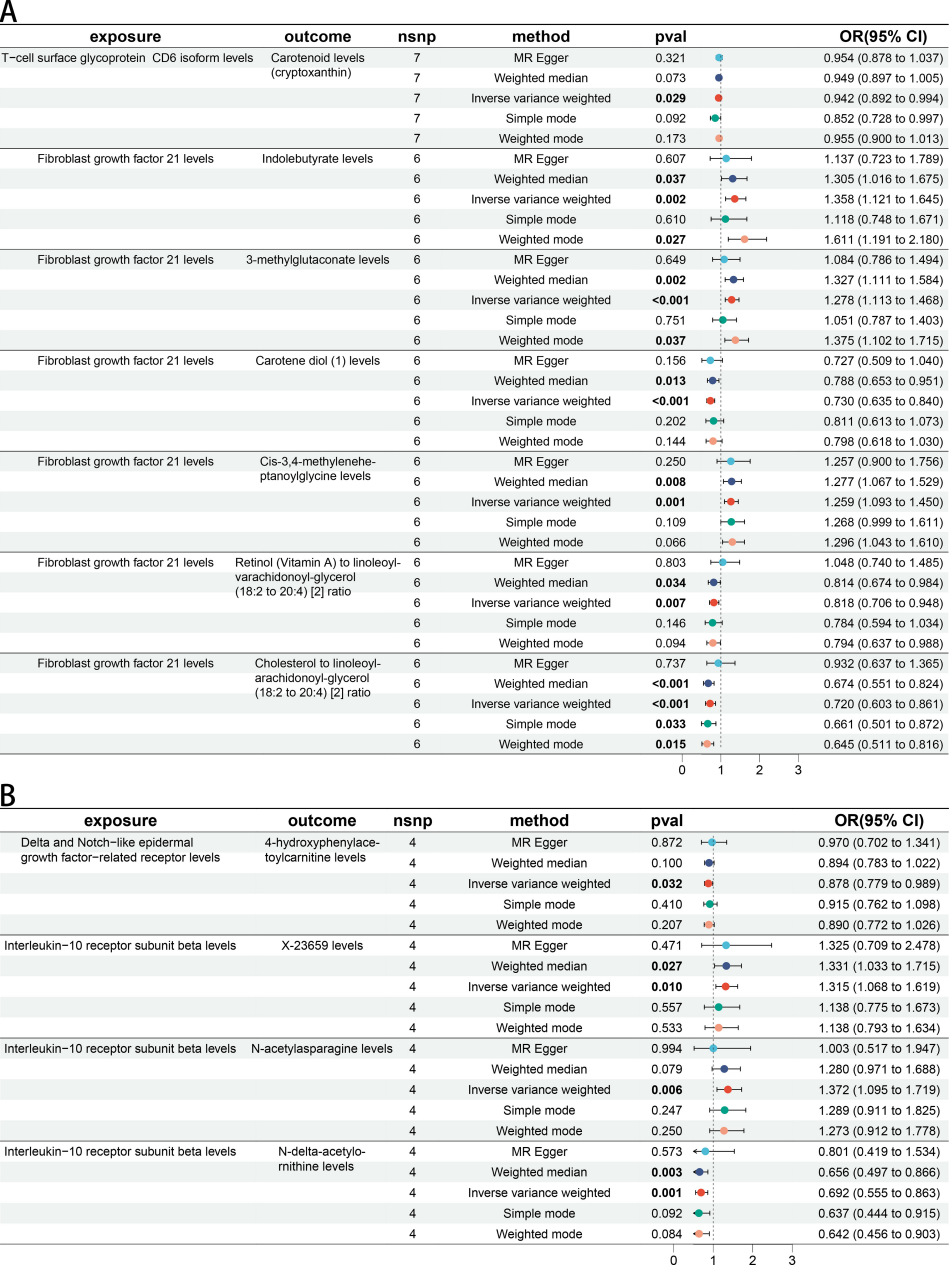
The causal association between CIPs and BMs. **(A)** Forest plot of casual effect between *Keloid scar* related CIPs and BMs. **(B)** Forest plot of casual effect between *Scar conditions and fibrosis of skin* related CIPs and BMs.

There were 30 BMs potentially associated with *keloid* (IVW *p* < 5 × 10^−2^) and 19 BMs potentially associated with *Scar conditions and fibrosis of skin* (IVW *p* < 5 × 10^−2^) (**Figures 3A, B**). More details could be found in **Supplementary Table 4**. In step 1 of two-step of MR model, we found that CD6 levels were negatively related to Carotenoid levels (cryptoxanthin) (OR 0.942 [95% CI 0.892, 0.994]). FGF21 levels were positively related with Indolebutyrate levels (OR 1.358 [95% CI 1.121, 1.645]), 3-methylglutaconate levels (OR 1.278 [95% CI 1.113, 1.468]) and Cis-3,4-methyleneheptanoylglycine levels (OR 1.259 [95% CI 1.093, 1.450]). FGF21 levels were negatively related with Carotene diol (1) levels (OR 0.730 [95% CI 0.635, 0.840]), Retinol (Vitamin A) to linoleoyl-arachidonoyl-glycerol ratio (OR 0.818 [95% CI 0.706, 0.948]) and Cholesterol to linoleoyl-arachidonoyl-glycerol ratio (OR 0.720 [95% CI 0.603, 0.861]). All the IVW P-values were < 5 × 10^−2^ (**Figure 4A**). DNER levels were negatively related to 4-hydroxyphenylacetoylcarnitine levels (OR 0.878 [95% CI 0.779, 0.989]). IL10RB levels were positively related to X-23659 levels (OR 1.315 [95% CI 1.068, 1.619]) and N-acetylasparagine levels (OR 1.372 [95% CI 1.095, 1.719]). IL10RB levels were negatively related to N-delta-acetylornithine levels (OR 0.692 [95% CI 0.555, 0.863]). All the IVW P-values were < 5 × 10^−2^ (**Figure 4B**). More details could be found in **Supplementary Table 5**.

### 3.4 Causal effects of the BMs on skin fibrosis

**Figure 5:**
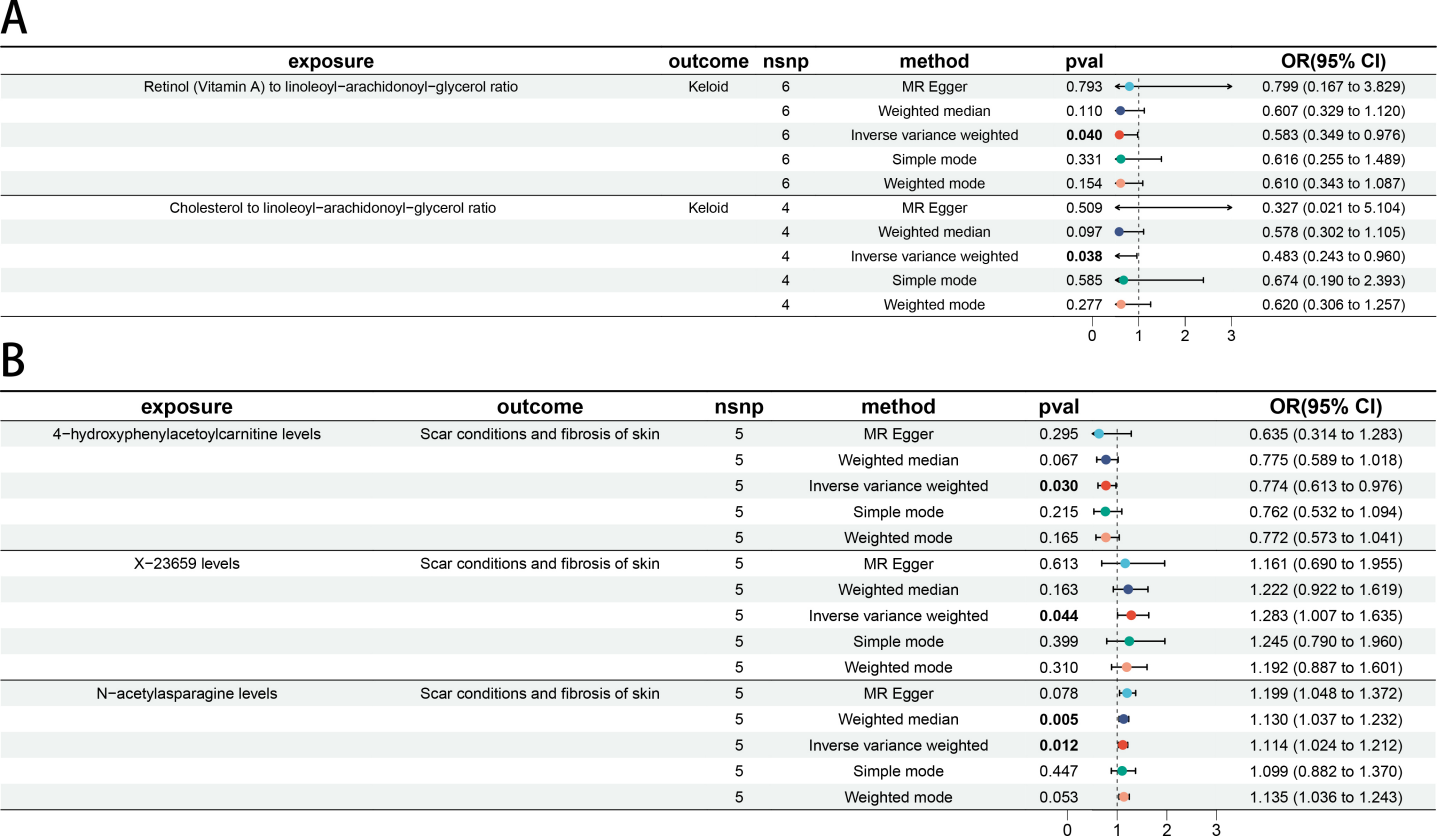
The causal association between BMs and skin fibrosis. **(A)** Forest plot of casual effect between *Keloid scar* related BMs and *Keloid scar*. **(B)** Forest plot of casual effect between *Scar conditions and fibrosis of skin* related BMs and *Scar conditions and fibrosis of skin*.

In step 2 of two-step of MR model, Retinol (Vitamin A) to linoleoyl-arachidonoyl-glycerol ratio was negatively related to *Keloid* (OR 0.583 [95% CI 0.349, 0.976]). Cholesterol to linoleoyl-arachidonoyl-glycerol ratio was negatively related to *Keloid* (OR 0.483 [95% CI 0.243, 0.960]) (**Figure 5A**). 4-hydroxyphenylacetoylcarnitine levels were negatively related to *Scar conditions and fibrosis of skin* (OR 0.774 [95% CI 0.613, 0.976]). X-23659 levels were positively related to *Scar conditions and fibrosis of skin* (OR 1.283 [95% CI 1.007, 1.635]). N-acetylasparagine levels were positively related to *Scar conditions and fibrosis of skin* (OR 1.114 [95% CI 1.024, 1.212]). All P-values were < 5 × 10^−2^ (**Figure 5B**). More details could be found in **Supplementary Table 6**.

### 3.5 Mediation analysis

**Figure 6:**
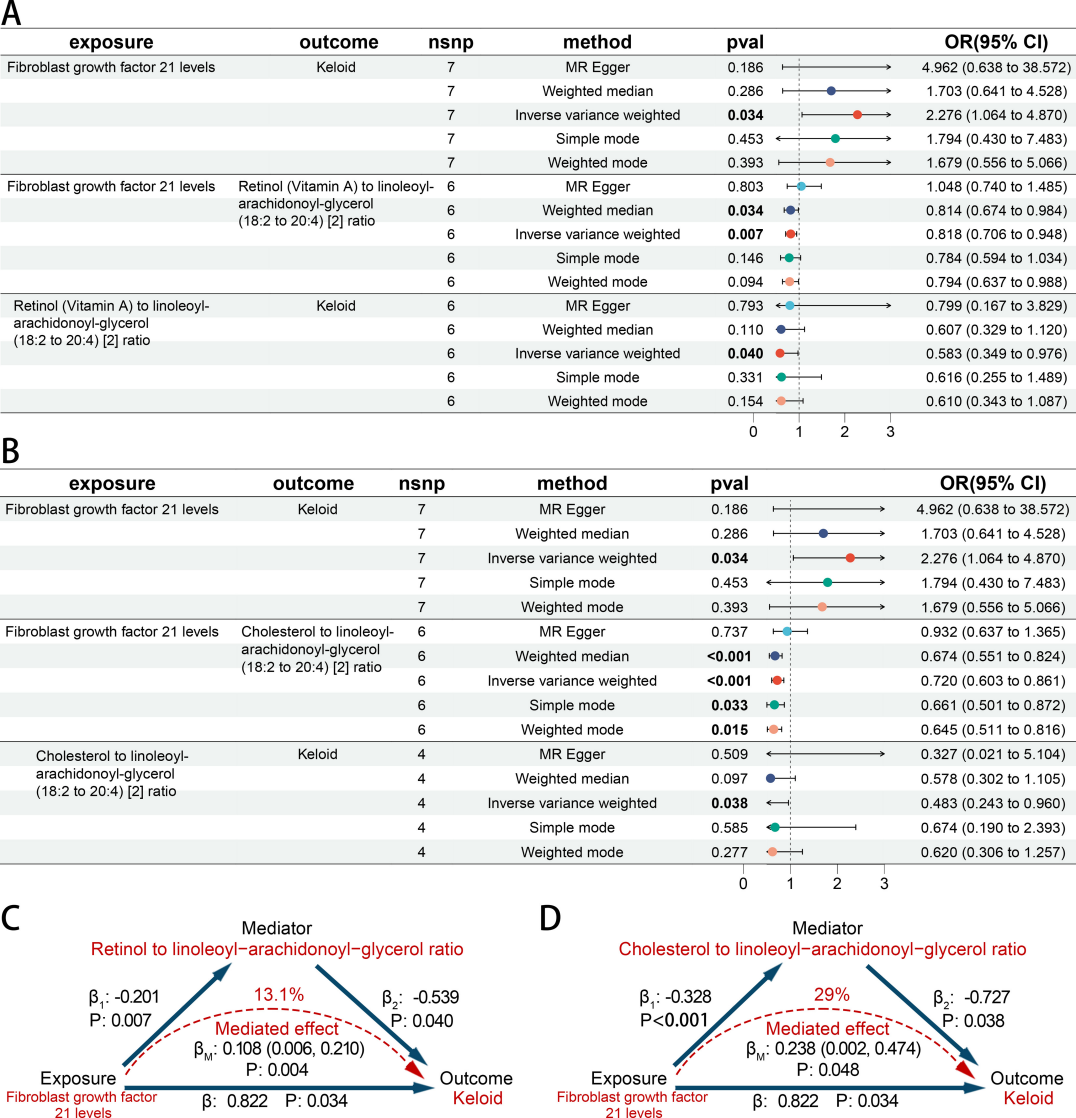
The causal association among CIPs, BMs and skin fibrosis. **(A, B)** Forest plot of casual effect among CIPs, BMs and *keloid scar*. **(C, D)** The effect and causal pathways among CIPs, BMs and *keloid scar*.

Using the product of coefficients method, we calculated the indirect mediation effect based on the results of the two-step MR model. FGF21 levels were positively associated with *Keloid* (OR 2.276 [95% CI 1.064, 4.870]). It was found that there is a negative association between FGF21 levels and Retinol (Vitamin A) to linoleoyl-arachidonoyl-glycerol ratio (OR 0.818 [95% CI 0.706, 0.948]). Retinol (Vitamin A) to linoleoyl-arachidonoyl-glycerol ratio was negatively related to *Keloid* (OR 0.583 [95% CI 0.349, 0.976]) (**Figure 6A**). It was found that there is a negative association between FGF21 levels and Cholesterol to linoleoyl-arachidonoyl-glycerol ratio (OR 0.720 [95% CI 0.603, 0.861]). Cholesterol to linoleoyl-arachidonoyl-glycerol ratio was negatively related to *Keloid* (OR 0.483 [95% CI 0.243, 0.960]) (**Figure 6B**). Then mediated effect analysis showed evidence of the mediated effect of FGF21 on *Keloid* through Retinol (Vitamin A) to linoleoyl-arachidonoyl-glycerol ratio (β_M_ 0.108 [95% CI 0.006, 0.210], *p* = 0.004) and Cholesterol to linoleoyl-arachidonoyl-glycerol ratio (β_M_ 0.238 [95% CI 0.002, 0.474], *p* = 0.048) with a mediated proportion of 13.1% and 29% of the total effect, respectively (**Figures 6C, D**).

Frost plots were also exhibited for the other traits, but their credible mediated effect and proportion could not be calculated (**Supplementary Figure S5**).

## 4 Discussion

The results of MR analyses revealed a causal association between CIPs and skin fibrosis. The levels of CD6, DNER, and IL10RB were negatively related to skin fibrosis while FGF21 was associated with an increased risk of skin fibrosis. According to mediation analysis, we found the evidence of mediated effect of FGF21 on skin fibrosis through Retinol (Vitamin A) to linoleoyl-arachidonoyl-glycerol ratio and Cholesterol to linoleoyl-arachidonoyl-glycerol ratio.

The role of inflammatory factor is crucial in the development of many diseases and is often targeted for therapeutic intervention. For example, the presence of ILR supports the maintenance of a CD8^+^ T cell population that sustains anti-tumor immunity (29). CD6 was identified as a therapeutic target of lupus nephritis (30). Changes in the expression levels of inflammatory factors, such as TGFB, can be detected in skin fibrotic tissues (8). Early diagnosis and treatment help prevent irreversible sequelae, such as cutaneous and subcutaneous atrophy. However, obtaining information for prevention and early diagnosis without tissue sampling or resection can be challenging. While CCL18 has been reported as a biomarker of skin fibrosis as an inflammation-related plasma protein, the investigation of CIPs in this area is still ongoing (9). There is a lack of causal association between CIPs and skin fibrosis. It is unclear whether CIPs is solely an index of skin fibrosis, or it can be considered as a target for diagnosis and treatment. For example, c-reactive protein (CRP) was considered as a biomarker of sepsis and COVID-19 disease progression (31, 32). However, it may not be appropriate to consider CRP as a therapeutic target for these diseases. In the present study, it was demonstrated that CD6, DNER, FGF21 and IL10RB exhibited a casual association with skin fibrosis. This finding suggests that these CIPs could be considered as potential therapeutic targets as well as biomarker. The CD6 lymphocyte receptor has been implicated in psoriasis, a chronic inflammatory skin disease (33). Targeting CD6 is an effective and well-tolerated novel biological therapy in moderate to severe psoriasis (34). In addition, CD6 was reported to be able to delay skin senescence induced by ultraviolet radiation b radiation (35). As we know, skin fibrosis is a typical aging-related pathological process (36). Modulating cellular senescence can inhibit fibrosis (37, 38). Further exploration of CD6 function provides insights into novel targets for addressing skin fibrosis. FGF21 was reported to be positively related to senescent cell accumulation at systemic and cellular levels (39). Besides, FGF21 is upregulated in tissue and can promote migration and differentiation of epidermal cells during wound healing (40). The disorder of plasma FGF21 may have an influence in disturbance of the tissue repair mechanism, which can ultimately result in skin fibrosis. What’s more, IL10RB has been demonstrated to limit liver fibrosis by inducing stellate cell senescence upon binding to IL22 (41). IL10 inhibits autophagy in hypertrophic scar fibroblasts via IL10-IL10R-STAT3 pathway which is helpful for treating skin fibrosis (42). The function of DNER is still unclear in fibrosis. These CIPs could be potential therapeutic targets for preventing and diagnosing skin fibrosis early. Improving our understanding of CIPs is expected to improve patients’ quality of life and provide insights into novel targets for addressing skin fibrosis.

Targeting CIPs may be a difficult task. We can focus on substances that are easy to regulate to intervene in the effect of CIPs on skin fibrosis. BMs is currently a focus and hotspot of research as it plays a crucial role in both tumor and non-tumor diseases (11, 43). BMs levels can be regulated by many factors, such as diet and lifestyle (10). Given the reported correlation between BMs and CIPs, regulating BMs may be a viable strategy (13, 14). There is evidence of the mediated effect of CIPs on skin fibrosis through BMs in our study. According to mediation analysis, both Retinol (Vitamin A) to linoleoyl-arachidonoyl-glycerol ratio and Cholesterol to linoleoyl-arachidonoyl-glycerol ratio mediated the effect of CIPs on skin fibrosis. The mediated effect of Retinol (Vitamin A) to linoleoyl-arachidonoyl-glycerol ratio was 0.108, and the mediated proportion was13.1%. In comparison, the mediated effect of Cholesterol to linoleoyl-arachidonoyl-glycerol ratio was 0.238 and the mediated proportion was 29%. Retinol has been shown to promote various anti-aging benefits for the skin (44, 45). In addition, it has been documented that retinol has an influence on the specification and differentiation of fibroblasts, indicating an anti-fibrotic effect (46). Cholesterol is a vital skin barrier lipid that plays a crucial role in maintaining skin homeostasis. The impairment of the skin barrier and disruption of skin homeostasis, which can lead to leaky epithelia and disease (47, 48). A cholesterol deficiency can lead to skin damage which may cause skin fibrosis (2, 49). It is important to maintain adequate levels of cholesterol for healthy skin. However, excessive intake of cholesterol can harm organs such as the liver and cardiovascular system (50). Compared with cholesterol, it is a better choice to promote the intake of retinol when intervening in the effect of FGF21 on skin fibrosis through retinol. Concurrently, it is also necessary to maintain the equilibrium of cholesterol levels within the plasma. Moreover, a more profound understanding of the interactions between the CIPs and BMs may facilitate the development of more efficacious treatments.

However, there are some limitations in our study. Caution should be exercised when interpreting these results as the study was analyzed at the genetic level. As the study individuals were predominantly of European ancestry, it is worth investigating whether these results apply to other ethnic groups, despite the large sample size (20). Furthermore, in the absence of a sufficiently extensive CIP dataset, it becomes challenging to conduct analyses encompassing all categories of CIPs such as CD8, CCL18 and CRP. Consequently, the present study is unable to explore the casual association between these CIPs and skin fibrosis, as well as whether BMs act as a mediating role in it. Large MR analyses were conducted on 1400 BMs traits, 91 CIPs traits, and two skin fibrosis traits. It is difficult to perform Bonferroni correction to obtain statistically significant results. Therefore, caution should be exercised when interpreting results with IVW-derived P values less than 5 × 10^−2^.

## 5 Conclusions

Skin fibrosis is a chronic dermatological condition characterized by a disruption of skin homeostasis. Early clinical manifestations of skin fibrosis are characterized by the expansion of sclerotic lesions. Early screening and treatment can prevent extensive skin involvement and avoid irreversible sequelae such as contractures and severe atrophy. MR analyses were performed to ascertain the causal association between the CIPs, CD6, FGF21, IL1RB, and DNER, and skin fibrosis. The results suggested that CIPs could be a potential target for skin fibrosis. Mediated effect analysis showed evidence of the mediated effect of FGF21 on the skin fibrosis through Retinol (Vitamin A) to linoleoyl-arachidonoyl-glycerol ratio and Cholesterol to linoleoyl-arachidonoyl-glycerol ratio. The analysis showed that BMs mediated the effect of CIPs on skin fibrosis.

It is essential to investigate the role of specific CIPs in dermatological conditions for precise and personalized treatments. Compare with tissue sampling or resection, the level of inflammation-related plasma protein could be detected in a rapid and efficient manner for early screening and diagnosis. Besides, the targeting of both specific CIPs and BMs may provide novel therapeutic strategies for patients. By detecting the level of the specific CIPs, it is possible to evaluate the effect of treatment and modify the therapeutic strategy. Improving the understanding of the impact of CIPs on skin fibrosis has the potential to improve patients’ quality of life and facilitate the development of innovative strategies for skin fibrosis.

## Data Availability

The original contributions presented in the study are included in the article/**Supplementary Material**. Further inquiries can be directed to the corresponding authors.

## Supplementary material

Supplementary Figure 1 Leave-one-out plot, forest plot, funnel plot, and scatter plot for the causal association between CD6 levels and *keloid scar*.

Supplementary Figure 2 Leave-one-out plot, forest plot, funnel plot, and scatter plot for the causal association between FGF21 levels isoform levels and *keloid scar*.

Supplementary Figure 3 Leave-one-out plot, forest plot, funnel plot, and scatter plot for the causal association between DNER levels and *Scar conditions and fibrosis of skin*.

Supplementary Figure 4 Leave-one-out plot, forest plot, funnel plot, and scatter plot for the causal association between IL10RB levels and *Scar conditions and fibrosis of skin*.

Supplementary Figure 5 Forest plot of casual effect among CIPs, BMs and *Scar conditions and fibrosis of skin*.

Supplementary Table 1 The CIPs list and overview of the source of data.

Supplementary Table 2 SNPs associated with skin fibrosis (5 × 10^−8^).

Supplementary Table 3 MR and reverse MR analyses between 91 CIPs and skin fibrosis.

Supplementary Table 4 MR analysis between 1400 BMs and skin fibrosis.

Supplementary Table 5 MR and reverse MR analyses between CIPs and BMs.

Supplementary Table 6 MR and reverse MR analyses between BMs and skin fibrosis.
