# Supplementary figures and images for: Association and mediation between circulating inflammatory proteins and skin fibrosis
Source: Front Endocrinol (Lausanne). 2025 Mar 18;16:1416993. doi: 10.3389/fendo.2025.1416993 (PMC11958232; doi:10.3389/fendo.2025.1416993)

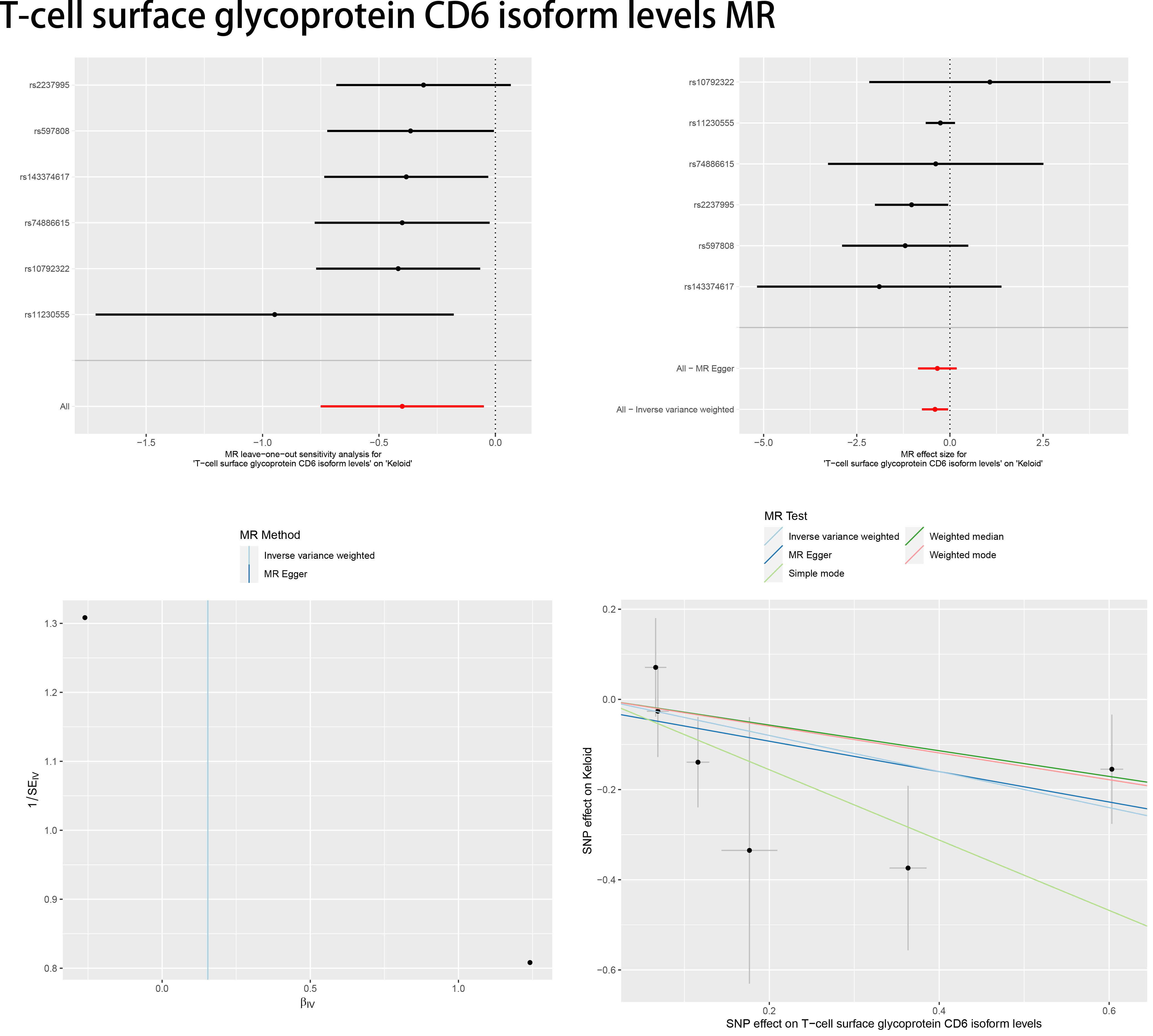

Supplement: Supplementary Figure 1 — Leave-one-out plot, forest plot, funnel plot, and scatter plot for the causal association between CD6 levels and keloid scar. [file Image1.jpeg]

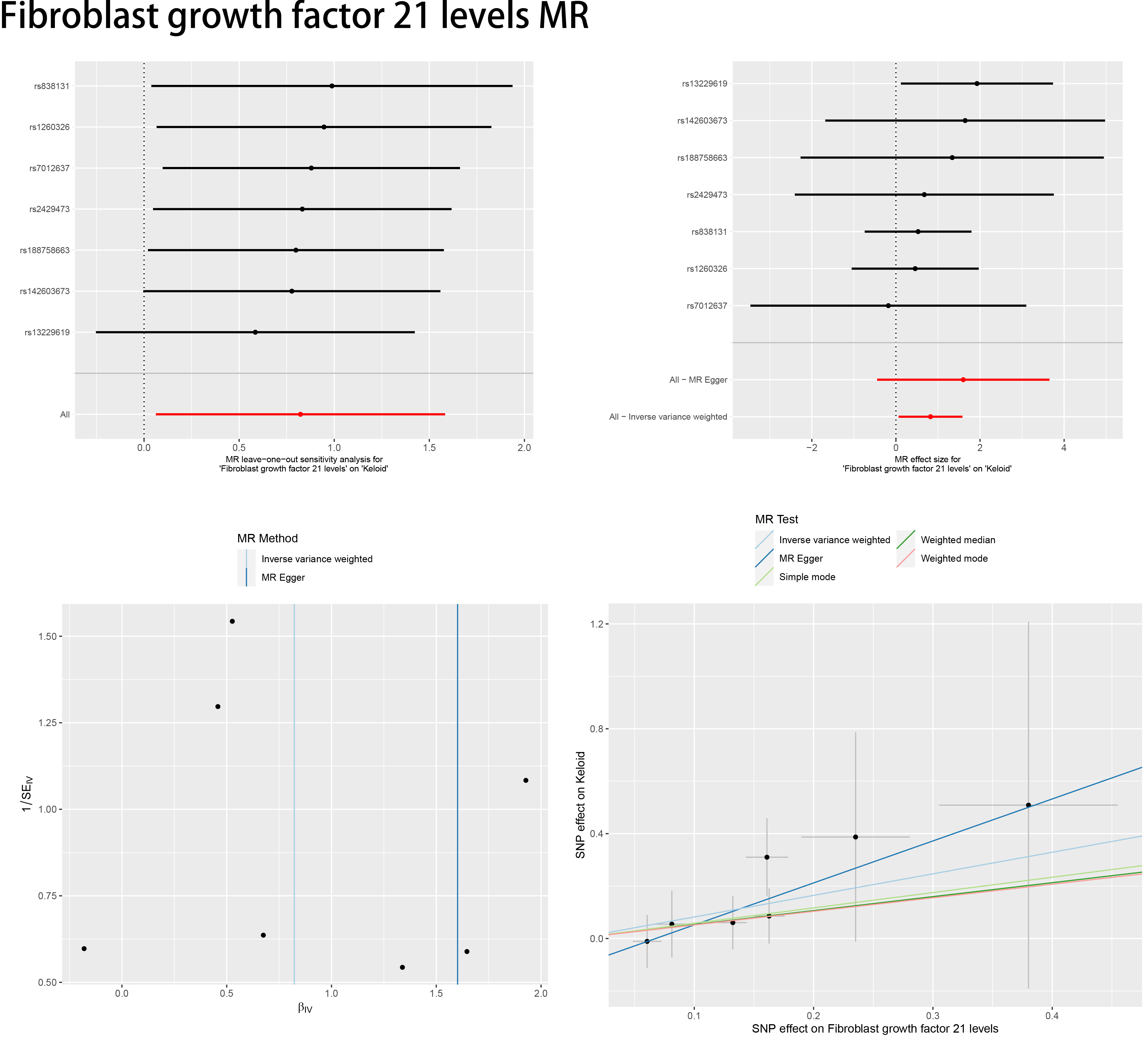

Supplement: Supplementary Figure 2 — Leave-one-out plot, forest plot, funnel plot, and scatter plot for the causal association between FGF21 levels isoform levels and keloid scar. [file Image2.jpeg]

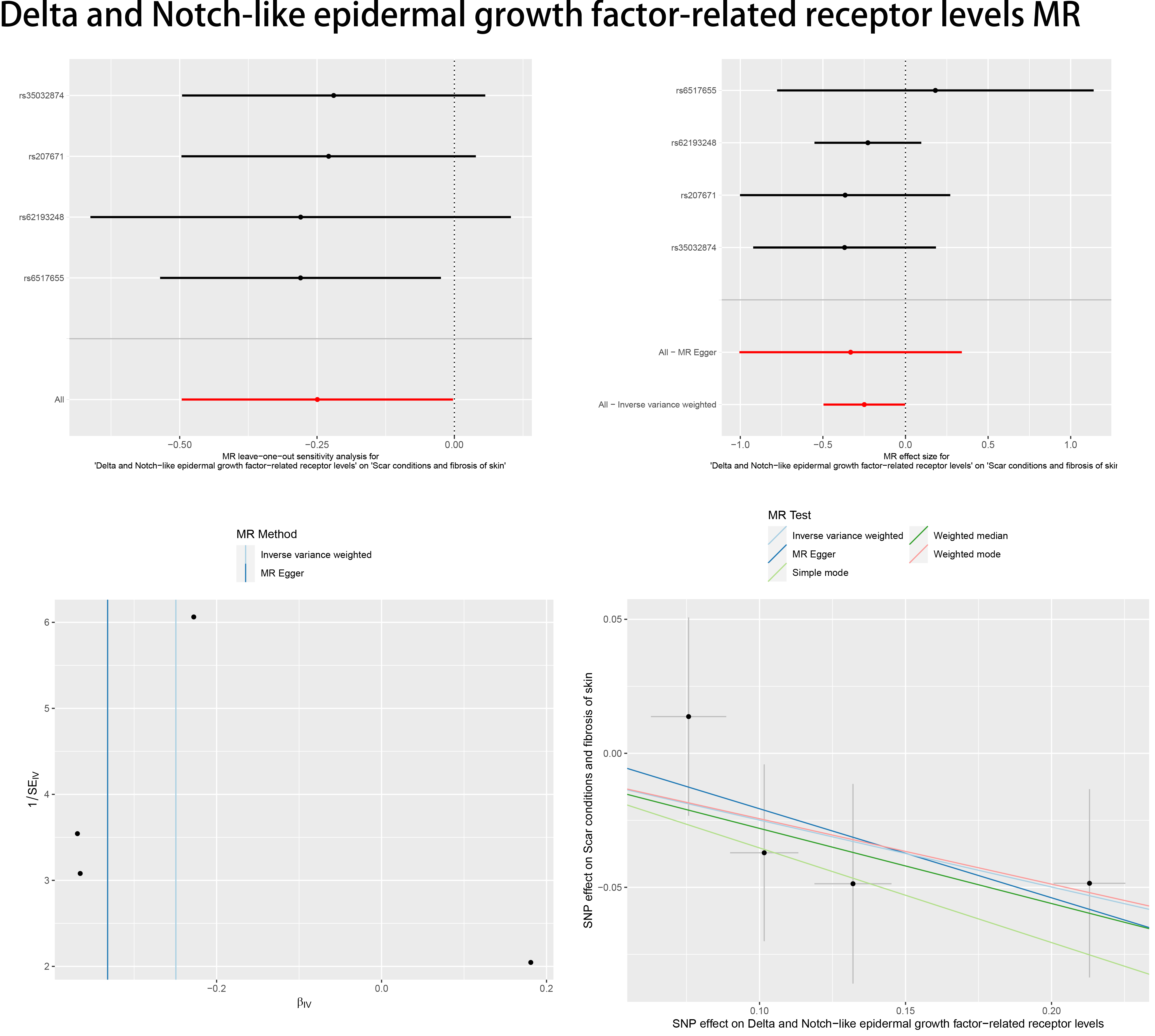

Supplement: Supplementary Figure 3 — Leave-one-out plot, forest plot, funnel plot, and scatter plot for the causal association between DNER levels and Scar conditions and fibrosis of skin. [file Image3.jpeg]

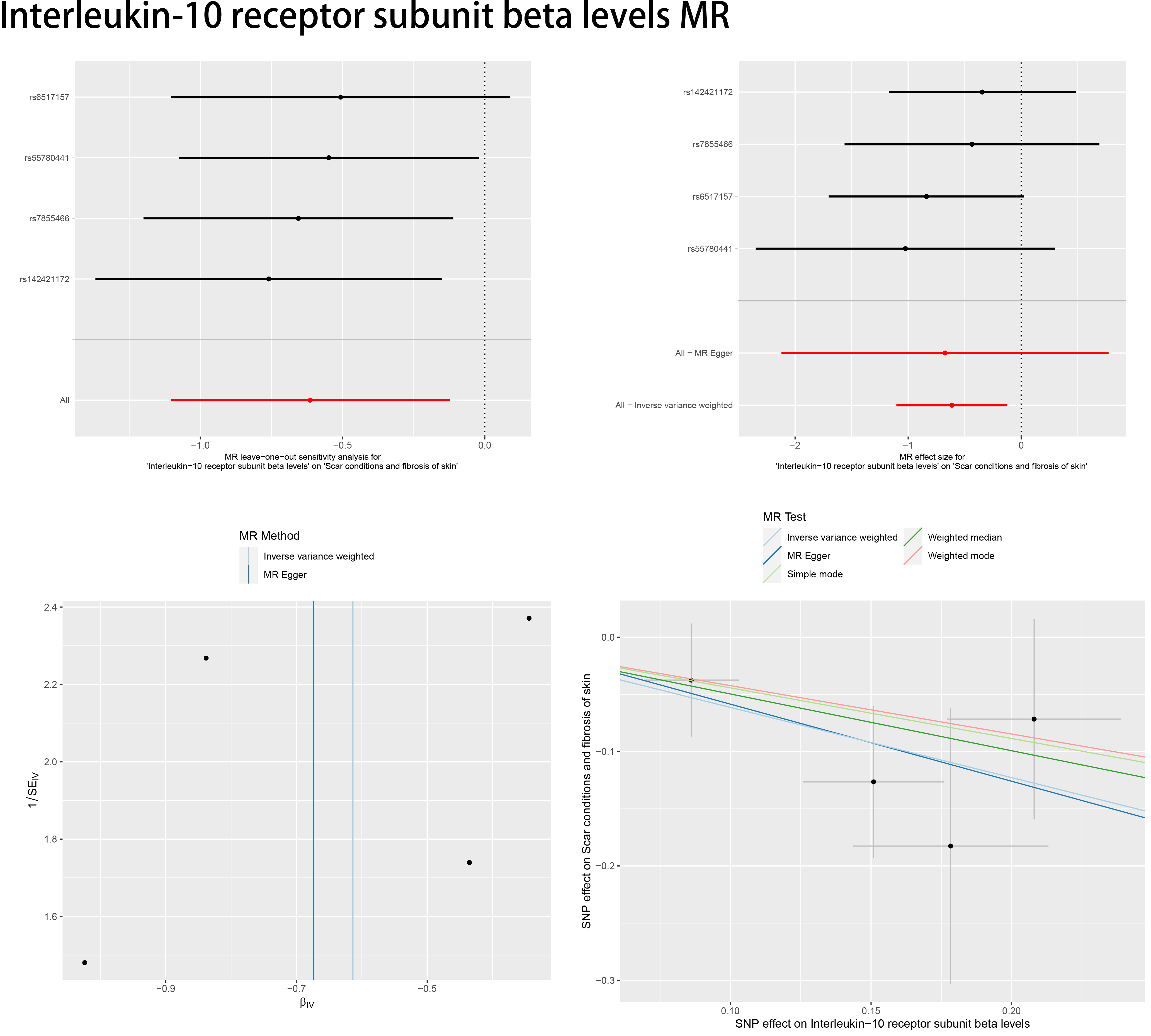

Supplement: Supplementary Figure 4 — Leave-one-out plot, forest plot, funnel plot, and scatter plot for the causal association between IL10RB levels and Scar conditions and fibrosis of skin. [file Image4.jpeg]

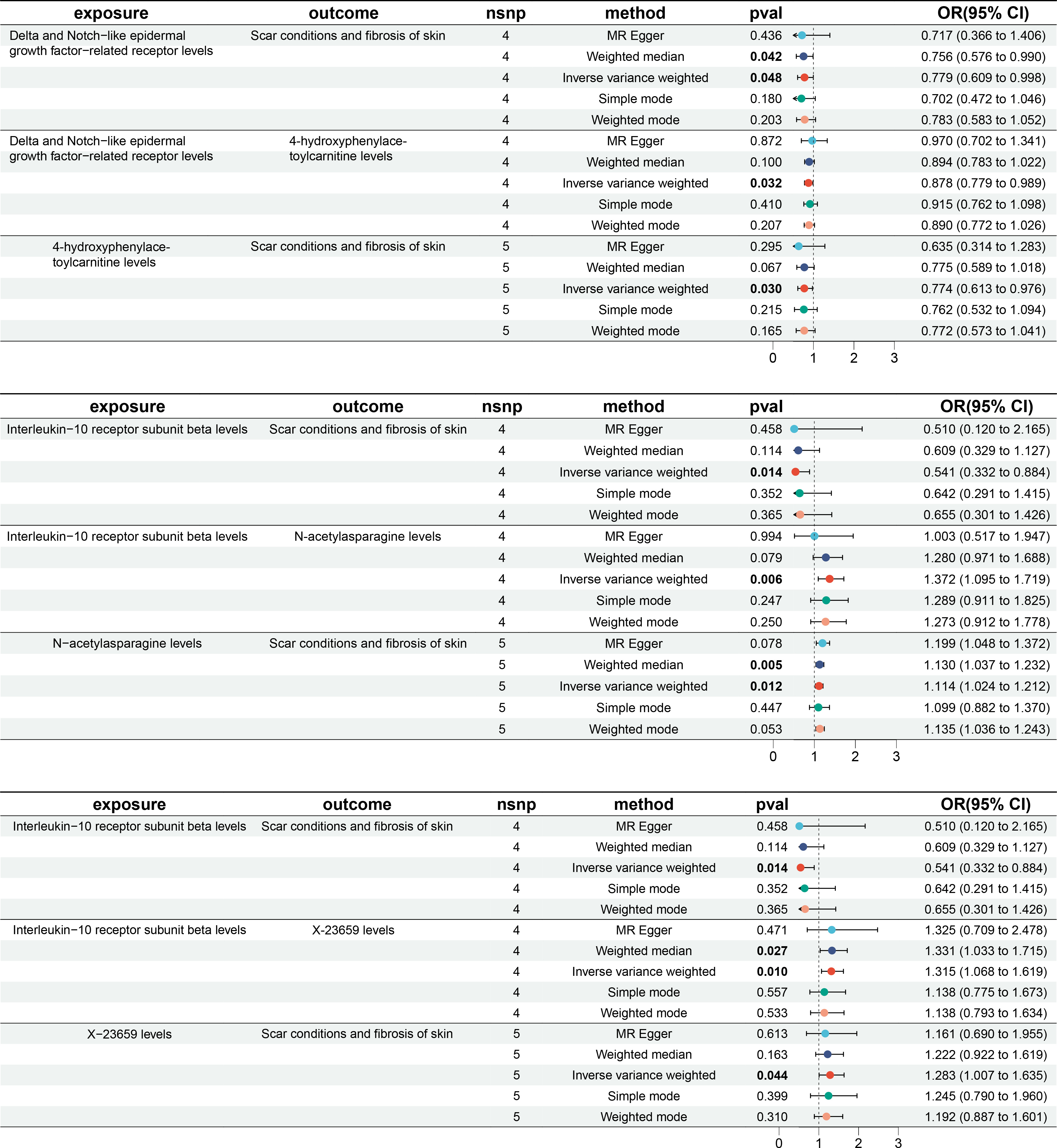

Supplement: Supplementary Figure 5 — Forest plot of casual effect among CIPs, BMs and Scar conditions and fibrosis of skin. [file Image5.jpeg]
